# Editorial: Artificial Intelligence in Environmental Microbiology

**DOI:** 10.3389/fmicb.2022.944242

**Published:** 2022-06-13

**Authors:** Mohammad-Hossein Sarrafzadeh, Seyed Soheil Mansouri, Javad Zahiri, Solange I. Mussatto, Hashem Asgharnejad

**Affiliations:** ^1^School of Chemical Engineering, College of Engineering, University of Tehran, Tehran, Iran; ^2^Department of Chemical and Biochemical Engineering, Technical University of Denmark, Kongens Lyngby, Denmark; ^3^Department of Neuroscience, University of California, San Diego, San Diego, CA, United States; ^4^Department of Pediatrics, University of California, San Diego, San Diego, CA, United States; ^5^Department of Biotechnology and Biomedicine, Technical University of Denmark, Kongens Lyngby, Denmark; ^6^Department of Civil, Geological, and Mining Engineering, Polytechnique Montréal, Montreal, QC, Canada

**Keywords:** artificial intelligence, deep learning, machine learning, environmental microbiology, predictive modeling, image analysis, metagenomics

Perhaps twenty-first century is so called “Digital Era” since digitalization and artificial intelligence (AI) is finding its way into every aspect of human life. Nowadays, AI-based approaches are gaining a lot of traction as components of research and development in different scientific and technological fields. One of the areas that is experiencing a digital revolution is environmental microbiology, which is the science of studying the interactions between the microorganisms and the environment and their mutual impacts (Pepper et al., 2011). Approaches such as machine learning (ML), deep learning (DL), image processing, pattern recognition and internet of things (IoT) are being widely implemented in this field in all aspects from theoretical development and identification to process monitoring and optimization (Asgharnejad and Sarrafzadeh, 2020; Gargalo et al., 2020; Asgharnejad et al., 2021). Outbreak of the global challenge of COVID-19 pandemic during the last 2 years and the huge impacts of this virus on socioeconomic infrastructures has also highlighted the necessity of innovative approaches for controlling and monitoring microbial communities in the environment. This special issue provides a platform for gathering the most recent advances in the fields of environmental microbiology from the perspective of AI. It includes 10 scientific papers (six original research articles, two mini-reviews and two reviews) that cover a wide range of AI approaches including ML, DL, and image processing. Two of these papers are specifically focused on using AI for diagnosis and tackling the SARS-CoV-2 virus, which is the species causing COVID-19 and, in this regard, the current Research Topic can be a reference for ongoing research on the edge of science to overcome the pandemic and prevent future such catastrophic outbursts.

Moreover, AI can be used to diagnose and find effective treatments for microbial-risen diseases. Previous studies have shown that oral microbiota has a close relation with different types of cancer. Wen et al. have studied the possible relation between the oral microbiota and gliomas, which are the most prevalent form of primary malignant brain tumors. They conducted an association rule mining algorithm to find the relation between the microbiota existed in the saliva of a compound sample containing 35 patients diagnosed with high-grade and low-grade glioma and 24 control samples. The results of their study determined the oral microbiota features and gene functions that were associated with glioma malignancy, which is a great achievement in terms of cancer therapy.

Zhang et al., conducted a literature review on how DL can accelerate the procedure of drug discovery to tackle Severe Acute Respiratory Syndrome Coronavirus 2, which is globally known as COVID-19. Besides improving the efficacy of antimicrobial screening against a wide range of pathogens, DL has shown potential to reliably identify drug candidates against newly-emerged diseases such as COVID-19 and a number of drugs including Atazanavir, Remdesivir, Kaletra, Enalaprilat, Venetoclax, and Posaconazole have been proposed to be effective based on applying DL algorithm on the datasets of genetic and protein types of SARS-CoV-2. Pi et al., also applied Heterogeneous Multi-Attention Graph Neural Network with the objective of drug prediction for COVID-19 and two drugs were successfully predicted and verified by their model.

DL can also be used to train a model for identifying the substrate secretion mechanism in Gram-negative bacteria. Chen et al. followed this approach using the sequence-based non-RTX-motif features and combined it into a tri-layer stacking model, T1SEstacker, which predicted the RTX proteins accurately. This model can accurately estimate the various substrate proteins being secreted through the two bacterial cell membranes by one step (classical) or two steps (non-classical) into extracellular environment.

Image datasets of environmental microorganisms such as EMDS-6 are powerful tools for diagnosis and classifications of newly discovered microorganisms. In the research carried out by Zhao, Li, Rahaman, Xu, Yang, et al., a comparative study was conducted of DL methods of classification using the EMDS-6 image dataset. The authors compared 21 DL-based methods of image classification including direct classification, imbalanced training, and hyper-parameters tuning. Zhao, Li, Rahaman, Xu, Ma, et al. also used the classic algorithms of image processing such as image denoising, image segmentation, and object detection to analyze the EMDS-6 and its potential for being used to evaluate the performance of image processing algorithms.

Metagenomics is a revolutionary field that burst fundamental changes in environmental microbiology, which allows the characterization of all microorganisms in a sequencing experiment. To distinguish the microbes in terms of taxonomy and biological activity, the sequenced reads must essentially be associated with known genomes/genes. However, current association methods are inadequate in terms of rapidity and also accuracy, especially when detecting bacterial species or in specific cases such as virus, plasmids, and gene detection. Machine and deep learning methods can use the newly reconstructed genomes by metagenomics as models and be a platform for association. Mathieu et al. tried to assess the different machine learning based methods and their efficiency to enhance the annotation of metagenomic sequences.

The application of metagenomics data can be expanded to clean and safe drinking water systems. Mahajna et al. reviewed the literature on what kind of occupancy-abundance patterns are exhibited in the drinking water microbiome, how the drinking water microbiome evolves both spatially and temporally, and how different microbial communities can co-exist in the drinking water environment. They also evaluated the potential role of AI in addressing the predictive and mechanistic questions in this field.

However, applications of AI are not limited to human health and genetics, but they can be expanded to environmental protection areas as well, especially in hardly controllable environments such as aquacultures. Zhai et al. used AI to study the bacterial biofilms on the metallic alloys at 5,700 m depth undersea. They derived the sequencing data of the microbial communities of the biofilms and applied big data analytics methods to study the dataset and compare the microbial composition of the biofilms on different alloy surfaces. McElhinney et al. also reviewed the capability of ML algorithms for analyzing the big microbiological datasets produced as a result to the advent of microbial omics. The authors provided a briefing for ML, highlighting the concept of retaining biological sample information for supervised ML, and reviewed the state-of-the-art of ML-driven microbial ecology.

In this Research Topic, cutting-edge scientific research works are gathered focused on applications of AI methods for identifying, monitoring, and analyzing environmental microorganisms and alleviating their hazardous impacts on human life. The gaps and challenges addressed in this Research Topic can be the hot topic of further studies in the future regarding the comprehension of what is going to be anticipated as a crucial concept for tackling the challenges in the area of environmental microbiology in the forthcoming years.

## Author Contributions

All authors have made a significant, equal, direct and intellectual contribution to the preparation of this editorial note, and approved it for publication.

## Conflict of Interest

The authors declare that the research was conducted in the absence of any commercial or financial relationships that could be construed as a potential conflict of interest.

## Publisher's Note

All claims expressed in this article are solely those of the authors and do not necessarily represent those of their affiliated organizations, or those of the publisher, the editors and the reviewers. Any product that may be evaluated in this article, or claim that may be made by its manufacturer, is not guaranteed or endorsed by the publisher.
